# Progress on theory of planned behavior research: advances in research synthesis and agenda for future research

**DOI:** 10.1007/s10865-024-00545-8

**Published:** 2025-01-20

**Authors:** Martin S. Hagger, Kyra Hamilton

**Affiliations:** 1 Department of Psychological Sciences, University of California, Merced, USA; 2 Health Sciences Research Institute, University of California, Merced, USA; 3 Faculty of Sport and Health Sciences, University of Jyväskylä, Jyväskylä, Finland; 4 School of Applied Psychology, Griffith University, Mount Gravatt, Australia; 5 Social and Health Psychology Behavioral Research for Prevention and Promotion (SHARPP) Lab, Department of Psychological Sciences, University of California, Merced, 5200 N. Lake Rd, Merced, CA 95343 USA

**Keywords:** Research synthesis, Social cognition theory, Behavior change intervention, Mechanisms of action, Mediation and moderation, Meta-analytic structural equation modelling

## Abstract

The theory of planned behavior is a social cognition theory that has been widely applied to identify the psychological determinants of intentions and behavior in health contexts. Our 2015 meta-analysis of theory applications in chronic illness contributed to a burgeoning evidence base comprising syntheses supporting theory predictions in health behavior. In this review, we identify limitations of prior meta-analyses of theory applications in health behavior and highlight salient evidence gaps, summarize how recent meta-analyses of the theory have addressed some of the limitations, outline outstanding research questions, and suggest future research syntheses, including those currently in progress, to resolve them. We point to recent and ongoing meta-analyses addressing theory hypotheses and assumptions not tested in previous syntheses, such as perceived behavioral control moderating effects and indirect effects of environmental (e.g., sociostructural variables) and intrapersonal (e.g., personality traits) determinants on health behavior mediated by theory constructs. We also highlight meta-analyses examining behavioral effects of constructs representing extended processes (e.g., habit, implicit cognition) in the context of the theory. Further, we summarize recent meta-analyses addressing directional and causal inferences in theory effects, including meta-analyses of longitudinal studies and experimental and intervention research. We also highlight attempts to test the mechanisms of action of interventions based on the theory including the change meta-analysis method and mediation analyses. We conclude by summarizing the advances that recent meta-analyses of the theory have made to the evidence base of health behavior determinants and interventions and highlighting suggestions for meta-analyses that will further progress the evidence base.

Despite epidemiological research reporting associations between regular participation in health promoting behaviors and avoidance of health risk behaviors (e.g., smoking, alcohol consumption) and adaptive health outcomes, particularly markedly lower chronic disease risk (e.g., cardiovascular disease, cancers, diabetes; Adams et al., 2019), a substantive proportion of the population does not meet guideline levels of health behavior participation necessary to confer benefits (Liu et al., 2016). State and federal health departments and advocates have, therefore, identified the development and promulgation of interventions aimed at promoting health behavior change as a key public health priority (Glanz & Bishop, 2010; Michie & West, 2013; Oldenburg et al., 2010). At the individual level, theorists and researchers have suggested that such interventions should be based on behavioral theory to optimize their efficacy and effectiveness (Kok et al., 2016; Michie et al., 2018; Prestwich et al., 2015). Such theories enable specification of potentially modifiable health behavior determinants that represent the psychological processes underpinning action and may serve as targets for change as a result of exposure to techniques adopted in interventions (Hagger et al., 2020a; Johnston et al., 2021; Sheeran et al., 2017). Research on the theoretical determinants of health behavior is expected, therefore, to provide formative evidence that may guide intervention (Hagger, 2025).

Social cognition theories, which assume individuals’ behavioral decisions are informed by their reasoned processing of social information, are a prominent class of theory that have been consistently adopted in health behavior determinants research (Conner & Norman, 2015). The theory of planned behavior, prototypical of the social cognition approach, is a well-specified, general theory purposed to predict social behavior (Ajzen, 1991). The theory predicts that individuals’ behavioral, normative, and capacity beliefs, captured by the attitude, subjective norm, and perceived behavioral control constructs, respectively, are key determinants of intentions to perform a target behavior in future, and intentions are hypothesized to mediate effects of these constructs on subsequent behavior (Hagger, 2019). The theory has been widely applied in health behavior determinants research – predictive studies have supported its hypotheses and its capacity to account for unique variance in multiple health behaviors across a range of contexts and populations (e.g., Ajzen, 2011; Ajzen & Schmidt, 2020; Hagger, 2019). Meta-analyses synthesizing evidence from hundreds of applications of the theory in health behavior contexts have provided converging evidence for its proposed effects and indicated that they hold across behaviors and other sociodemographic and methodological moderators (e.g., Albarracín et al., 2001; Hagger et al., 2002; Hamilton et al., 2020; McEachan et al., 2011; Rich et al., 2015).

As an example, we offer our 2015 meta-analysis of studies applying the theory to predict adherence to treatment for chronic illness prevention and management (Rich et al., 2015). Our findings indicated that the theory not only accounted for unique variance in intentions and behavioral adherence and lent support to independent effects of each theory construct on intention, and of intention on behavior, across eligible studies, but also confirmed a key mechanistic role of intention as a mediator of belief-behavior relations. Also important was our tests of candidate moderators of theory effects in this context that indicated consistency regardless of behavior (e.g., diet, exercise, self-care activities, medication adherence), behavioral measure type (self-report, non-self-report), and population type (e.g., socio-demographic variables). In sum, our analysis represents one of many relatively robust data points derived from meta-analyses of theory tests that support to its predictive validity in health behavior contexts.

In the intervening 10 years since the publication of our analysis, there have been a number of notable conceptual and methodological advances on the theory of planned behavior, and others of its class, in health behavior contexts. These advances have addressed some identified limitations in research that has applied it that have furthered knowledge on health behavior determinants and the mechanisms involved. The advances have also had important ramifications for informing health behavior change interventions. In the current article, we provide a selective summary of salient conceptual and methodological advances in research applying the theory in health behavior contexts with a focus on meta-analytic syntheses, outline the contribution these advances have made to the furtherment of knowledge on health behavior determinants and health behavior change interventions, and discuss some of the most pertinent research questions arising from this evidence that should set an agenda for future research inquiry in this domain. We begin by identifying some conceptual shortcomings of prior research syntheses of the theory in health behavior contexts: (a) failure to test some central theory predictions such as perceived behavioral control moderating effects and conceptually-related hypotheses such as construct correspondence and patterns of effects of indirect and direct measures of theory constructs on intention and behavior; (b) lack of account for the effects of variables representing the broader environmental and intrapersonal determinants of health behavior; and (c) failure to consider the unique role of constructs representing other salient processes likely implicated in health behavior participation such as non-conscious or implicit processes. Next, we focus on some methodological issues that delimit the inferences that can be made from research syntheses: (a) limitations relating to causal and directional inferences in theory effects; and (b) a lack of account for the mechanisms by which interventions based on the theory operate. In each case, we outline how recent meta-analyses of the theory address these shortcomings and how they have contributed to augmenting the evidence base of health behavior determinants based on the theory. We present the diagram in Fig. 1 as an accompaniment to our review, which illustrates some of issues identified and suggested directions for future research.

## Comprehensive tests and additional processes in research syntheses

Prior meta-analyses of research applying the theory of planned behavior in health behavior (e.g., Albarracín et al., 2001; Hagger et al., 2002; McEachan et al., 2011), including our 2015 analysis (Rich et al., 2015), have lent support for some of its key hypotheses, such as unique effects of its constructs on intentions and behavior, and the mediating role of intentions. These syntheses have done so by applying multivariate analyses to meta-analytically synthesized data that mirror those typically used in primary research testing the theory, such as multiple regression, path analysis, and structural equation modelling^1^. However, these tests have not typically synthesized and tested key hypotheses central to the theory including the role of perceived behavioral control and measurement correspondence as moderators of theory effects and effects of global and belief-based measures of theory constructs. Further, prior syntheses have not typically examined the role of broader environmental and dispositional variables and constructs, or effects of constructs that represent other processes implicated in health behavior enactment. In this section, we visit each of these issues in turn and outline how recent meta-analyses have sought to address these limitations, their contribution, and potential avenues for future research.

### Comprehensive tests of theory hypotheses

**Fig. 1:**
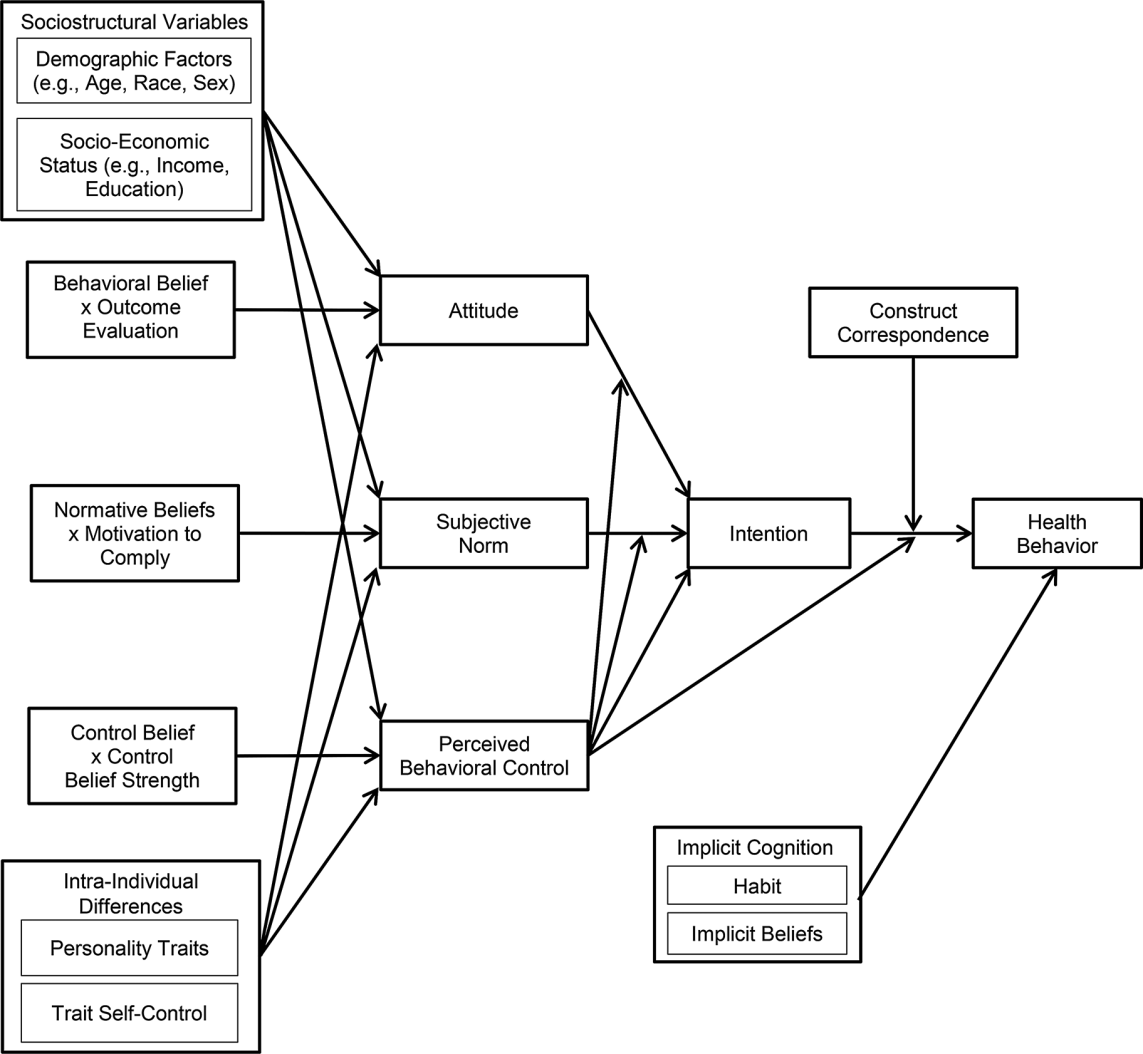
Schematic representation of theory of planned behavior constructs and its predicted effects augmented to include effects of additional variables, constructs, and moderators

The theory of planned behavior is, essentially, an integrated theory incorporating a key construct, perceived behavioral control, into its predecessor, the theory of reasoned action (Ajzen & Fishbein, 1980). Perceived behavioral control summarizes individuals’ beliefs with respect to their capacity to perform the behavior in future and has been explicitly aligned with other control-related constructs such as self-efficacy from social cognitive theory (Bandura, 1986). Ajzen (1991) proposed that effects of attitudes and subjective norms on intention, and intention on behavior, were conditional on an individual having high control over the behavior. As such, individuals with higher beliefs in their control over performing a given target behavior in future would cite fewer barriers and more facilitating conditions with respect to future behavioral performance. Under such conditions, individuals were expected to be more likely to form intentions to perform the behavior in accordance with their utility and normative beliefs, summarized in the attitude and subjective norm constructs, and would be more likely to follow through in enacting their intentions. Perceived behavioral control was, therefore, conceptualized as a moderator the attitude-intention, subjective norm-intention, and intention-behavior relationships. Further, to the extent that individuals reported their control over the behavior to be complete, attitude, subjective norm, and intention effects would be maximized, and the theory would, effectively, reduce to the theory of reasoned action. The perceived moderating effects are illustrated in Fig. 1 by the arrowed effects of perceived behavioral control on the effects of attitude and subjective norm on intention, and of intention on behavior.

The moderating effects were tested in Ajzen’s and colleagues’ early studies on the theory (e.g., Ajzen & Driver, 1992; Schifter & Ajzen, 1985). However, subsequent research tended to fixate on perceived behavioral control an indirect behavioral determinant mediated by intentions, and, when control perfectly aligned with behavior, a direct determinant. This means that fewer studies tested these moderating effects, and it was neglected in previous syntheses of research applying the theory in health behavior contexts (e.g., McEachan et al., 2011), including our previous analysis (Rich et al., 2015). Effectively, this rendered syntheses of research on the theory incomplete. The lack of meta-analyses of perceived behavioral control moderating effects could be attributed to the general lack of studies testing these effects, as well as a general tendency for researchers to not report sufficient data to provide a fit-for-purpose synthesis of these effects.

We recently addressed this evidence gap in a synthesis of existing primary data sets from two programs of research (Hagger et al., 2022). This enabled us to capitalize on the availability of sufficient data to test direct and interactive moderating effects of perceived behavioral control, consistent with Ajzen’s (1991) original hypotheses. Our research also employed recent advances in meta-analytic structural equation modeling which enable tests of moderation on individual effects in a model representing theory predictions (Jak & Cheung, 2020). Our analysis provided robust support for the moderation of the intention-behavior relationship by perceived behavioral control, consistent with the theory across 39 independent tests in ten health behaviors. Larger intention-behavior relations were observed among individuals reporting high control over their behavior. However, the analysis revealed much smaller moderating effects that were not distinguishable from the null for perceived behavioral control on attitude-intention and subjective norm-intention, so did not corroborate these predictions. Taken together, these findings provide the most comprehensive data yet in support of this hypothesized moderating effect for the intention-behavior relationship and moves the cumulative evidence supporting them in health behavior contexts beyond that provided in previous syntheses.

The moderating effect of perceived behavioral control aside, prior meta-analyses of the theory have tested effects of other conceptual and methodological moderator variables on theory effects in health behaviors. For example, meta-analyses have consistently tested the moderating role of measurement lag on intention-behavior relations (Hagger et al., 2002; Hamilton et al., 2020; McEachan et al., 2011). Findings have tended to confirm that a proximal (shorter) lag between measurement of theory constructs and behavior leads to larger intention-behavior effect sizes, consistent with the original theory hypothesis. Such moderator tests illustrate the power of meta-analyses to provide robust tests of theory predictions. However, prior meta-analyses of the theory have not tested a number of other key moderators rendering them somewhat incomplete and represent key gaps in current evidence syntheses of the theory.

A prominent untested moderator is measurement correspondence. Ajzen (1991) indicated that effective prediction of intentions and behavior in the theory is dependent on adoption of measures that capture the specific target behavior of interest precisely, consistent with the classic observation in attitude research that poor correspondence between attitude measures and behavior tends to attenuate or extinguish attitude-behavior associations (e.g., Wicker, 1969). Ajzen (1991) therefore proposed that measures of constructs such as attitudes and subjective norms should correspond with measures of intention and behavior in terms of target (i.e., the subject performing the behavior), action (i.e., the specific behavior of interest), context (i.e., the specific location or situation in which the behavior is to be performed), and time (i.e., the temporal period in which the behavior is to be performed). This moderating effect is illustrated by the direct arrowed effect on the intention-behavior effect in Fig. 1. Primary studies have indicated that measures that meet these correspondence standards lead to larger effects of the theory constructs on intentions and behavior (e.g., Ajzen & Timko, 1986). Future meta-analyses would do well to consider testing the moderating effects of measurement correspondence in studies applying the theory in health behavior contexts. This could be done by coding studies included in the meta-analysis according to the extent to which the adopted measures conformed to Ajzen’s correspondence standards. Accordingly, we are currently working on a large pre-registered meta-analysis of the theory applied in physical activity contexts to test this moderation effect (Simpson-Rojas & Hagger, 2021). We have developed a coding scheme that scores study measures according to the number of correspondence standards they meet. We will test the correspondence hypothesis by using scores as a moderator of averaged theory effects across studies. We expect to observe larger averaged effects of theory constructs on intention and behavior in studies adopting measures with high correspondence relative to those with lower correspondence.

A further fundamental set of predictions of the theory of planned behavior is the importance of salient beliefs as indirect indicators of the theory constructs. Ajzen (1991) proposed that the attitude, subjective norm, and perceived behavioral control constructs represent global summaries of specific sets of behavioral, normative, and control beliefs, respectively, that individuals hold regarding their future performance of the target behavior. In addition, individuals were also proposed to qualify each belief by a corresponding value, termed outcome evaluations, motivation to comply, and control belief strength, respectively, and applied an expectancy-value scoring model in which each belief measure was weighted by its corresponding value measure^2^. Salient beliefs were expected to vary across behavior, population, and context, and could be elicited through open-ended surveys administered in the population of interest. Most important, the belief-based constructs were expected to predict their respective global construct and each global construct expected to serve as a mediator of their respective belief-based construct effects on intentions and behavior. Although prior meta-analyses have supported associations between the belief-based and the global construct measures (Armitage & Conner, 2001), no meta-analysis to date has tested the proposed indirect effects. Previously, the relative dearth of studies incorporating measures of both belief-based and global constructs in health behavior contexts has been a key impediment to such an analysis. However, there now exists a critical mass of studies applying the theory in health behavior contexts that include measures of both construct forms to conduct this analysis. Accordingly, we propose to do so in our proposed updated meta-analysis of applications of the theory in physical activity contexts (Simpson-Rojas & Hagger, 2021). We expect the analysis will provide further formative evidence for interventions based on the theory given that it is the salient beliefs that should serve as primary targets for change in interventions (Ajzen & Schmidt, 2020).

### Environmental and intrapersonal determinants

A fundamental, although often overlooked, assumption of the theory of planned behavior is that its constructs should account for the effects of variables that represent sociostructural influences, and dispositional constructs that represent intrapersonal influences, on health intentions and behavior. Ajzen (1991) proposed that such constructs represent potentially-salient sources of information that individuals account for when estimating their beliefs with respect to their future behavioral performance. For example, sociostructural variables that represent barriers to performing the behavior (e.g., lack of facilities, low access to inexpensive healthy food options, poor access to healthcare) should be reflected in individuals’ estimates of their capacity to perform behavior, that is, their perceived behavioral control. Similarly, individuals who tend to endorse measures of intrapersonal dispositional constructs, such as the conscientiousness trait from the five-factor model of personality (Digman, 1990), are more likely to align their beliefs regarding the utility of the behavior toward servicing salient goals and are more likely to invest effort in pursuing them, which would be expected to be reflected in their attitudes and intentions, respectively. Consistent with this proposal, the theory constructs should mediate effects of sociostructural variables and intrapersonal constructs on behavior, providing a mechanistic explanation for observed associations between these factors and behavior. The proposed mediated effects are illustrated in Fig. 1 comprising the direct arrowed effects of the sociostructural (e.g., age, education, income, race, sex) and intrapersonal (e.g., personality, trait self-control) on the global measures of attitude, subjective norms, and perceived behavioral control, the direct effects of these constructs on intention, and the direct effect of intention on behavior.

Primary studies have lent support for hypotheses based on this assumption. For example, sociostructural variables such as indices of socio-economic status (e.g., income, education) and sociodemographic factors (e.g., age, sex, race; Godin et al., 2010; Orbell et al., 2017). Similarly, personality traits such as conscientiousness (Conner & Abraham, 2001), agreeableness and openness to experience (Bogg & Milad, 2020), and extroversion (Rhodes & Courneya, 2003), and sub-facets such as self-control (Conner et al., 2023; Hagger et al., 2019) have been shown to be indirectly related to health behavior mediated by the attitude, subjective norm, and perceived behavioral control constructs. However, it is only relatively recently that researchers have synthesized research testing these hypotheses in health behavior contexts. Such analyses are likely to have previously been precluded due to insufficient data reporting or availability – sociostructural variables, for example, have often considered mere covariates rather than as integral to the processes underpinning health intentions and behavior (Schüz, 2017). We recently capitalized in the proliferation of available studies encompassing these constructs, as well our own programs of research, to conduct meta-analyses corroborating primary research findings on the proposed mediation effects. Consistent with those prior findings, our analyses revealed averaged indirect effects of sociostructural variables such as sex and age (Hagger & Hamilton, 2021) and health literacy (McAnally & Hagger, 2023) on health behaviors. These analyses provide a mechanistic explanation for observed disparities in health behavior participation and, ultimately, health outcomes. For example, our meta-analysis indicated that relations between health literacy and health behavior participation were mediated by attitudes (McAnally & Hagger, 2023). Individuals with inadequate health literacy may not know of, or fully comprehend, health-behavior links. Limitations in knowledge or comprehension are likely reflected in beliefs in the utility of health behaviors to promote health outcomes, represented by the attitude construct. Overall, these analyses provide important corroboration of a key theory prediction.

However, there is considerable scope to extend evidence for this process-related effect in future research syntheses. For example, we are currently conducting pre-registered meta-analyses aimed at examining indirect effects of personality traits from the five-factor model (Hagger, 2018) and trait self-control (Hagger & Hamilton, 2024c; Primoceri et al., 2018) on health behaviors mediated by the theory constructs. Findings are expected to not only corroborate observed associations between these traits and health behavior, but provide further confirmation of the mediation hypotheses, particularly the constructs responsible for accounting for effects of these intrapersonal dispositional constructs. For example, relations between traits such as conscientiousness and self-control that reflect capacities to engage in goal-directed behavior likely positively orient individuals’ beliefs in capacity toward future health behavior participation. Such findings are important because although there is evidence that traits are malleable through intervention, effect sizes are small and may not be enduring (Roberts et al., 2017). By contrast, evidence suggests that the attitude, subjective norm, and perceived behavioral control constructs from the theory are more malleable and can be changed through the techniques used in behavior change interventions (Ajzen & Schmidt, 2020; Sheeran et al., 2016b). As a consequence, such research may provide some formative evidence of candidate constructs that could be targeted in studies evaluating the efficacy of theory-based behavior change interventions.

### Extending the theory to account for other processes

A prominent critique of the theory of planned behavior is the exclusive focus on constructs that represent reasoned, deliberative decision making. Such processes are captured by the intention-mediated effects of the attitude, subjective norms, and perceived behavioral control constructs on behavior that reflect utility, normative, and capacity considerations, respectively, with respect to performing a target health behavior in future. Theorists have suggested that such an approach neglects non-conscious, automatic, implicit processes that determine behavior beyond individuals’ attention with little or no deliberation (Hagger, 2016; Sheeran et al., 2013). As a consequence, researchers have proposed and tested integrated models that augment the theory of planned behavior with additional constructs that reflect the non-conscious processes implicated in health behavior (Hagger & Hamilton, 2020). These integrated models draw their inspiration from dual process models of cognition and social behavior (e.g., Deutsch & Strack, 2020). Such research has increased in intensity since our 2015 meta-analysis. Examples include studies that encompass measures of the habit construct (e.g., behavior frequency x context stability, response frequency, and self-report habit index measures; Verplanken et al., 1994; Verplanken & Orbell, 2003; Wood et al., 2002) and implicit cognition, such as implicit attitudes, identity, and motives (e.g., implicit association test, extrinsic affective Simon test; De Houwer, 2003; Greenwald et al., 1998). Studies testing integrated models have demonstrated that such constructs have unique effects on health behavior independent of intentions (e.g., Howell et al., 2016; Lindgren et al., 2015; Phipps et al., 2020). Critically, they have also identified conditions that determine when such measures tend to serve as the predominant determinant, such as when the behavior has been performed regularly and in the presence of stable contexts or cue in the case of habit (e.g., Ebert & Lin, 2024; Orbell & Verplanken, 2010), or when cognitive resources are low particularly in the face of high temptation or impulse to perform the behavior in the case of implicit cognition (e.g., Ellis et al., 2016; Friese et al., 2008). Consistent with this research, there is also intriguing evidence that interventions adopting techniques purposed to target these constructs leads to behavior change (e.g., Folkvord et al., 2016; Forscher et al., 2019; Kaushal et al., 2017). Studies such as these have advanced knowledge on health behavioral determinants and the processes involved (Sheeran et al., 2016).

Proliferation of research testing integrated models that extend the theory of planned behavior to encompass constructs representing non-conscious processes in health behavior contexts, has also inspired research syntheses to provide robust tests of these effects and evaluate their variability and generalizability. Here we offer our meta-analysis of research examining effects of measures of the habit construct as an example (Hagger et al., 2023). We synthesized studies reporting associations between habit measures, intentions, and behavior. We used meta-analysis to estimate the relative effects of habit and intention on behavior and effects of salient moderators such as likelihood of the behavior to be formed as a habit and behavioral complexity. Findings indicated that both habit and intentions predicted habit in multiple health behaviors across studies, and that averaged habit effects were larger in studies on behaviors likely to be formed as habits and behaviors low in complexity, corroborating habit theory. The analysis is the first to provide a comprehensive synthesis of these effects and provides important evidence in support of extending the theory to encompass habits as an independent behavioral determinant.

Prior meta-analyses have examined effects of implicit cognition such as implicit attitudes or identity on specific health behaviors (e.g., Rooke et al., 2008). This has been accompanied by a systematic review of research on implicit cognition across health behaviors (Rebar et al., 2016). However, there has been no comparable meta-analysis to date of research examining the relative effects of social cognition constructs such as those from the theory of planned behavior measured using explicit and implicit methods (e.g., explicit attitudes measured using survey measures and implicit attitudes measured using the implicit association test) on health behavior. To address this, we are currently conducting a large-scale pre-registered meta-analysis that reports independent effects of explicit and implicit attitudes on multiple behaviors, including health behaviors (Phipps et al., 2024). Preliminary findings indicate averaged independent effects of implicit and explicit attitudes on behavior, but no differences in the relative effects of the forms of attitude on behavior in behaviors that are more likely to be consciously controlled. However, a limitation of this analysis is that it will not account for intention effects and, therefore, precludes examination of the relative effects of the attitude forms and intentions on behavior consistent with the theory of planned behavior. We look to future research syntheses to investigate these effects.

## Inferences and mechanism tests in research syntheses

A frequent criticism levelled at research on theory-based determinants of health behavior, including studies based on the theory of planned behavior, is the preponderance of studies that adopt correlational designs (Hagger & Hamilton, 2021a; Hagger, 2025). These designs do not permit inference of direction or cause in theory effects, nor do they account for construct change over time. Given that theories such as the theory of planned behavior explicitly frame their predictions in causal terms (e.g., attitudes cause intentions, intentions cause behavior), direction and cause in effects from such studies are inferred from theory alone, not the data. Overreliance on correlational study designs has meant that the studies included in most meta-analyses of theory of planned behavior effects in health behavior, including our 2015 analysis (Rich et al., 2015), tend to be predominately or exclusively correlational in design. As a consequence, the same criticisms apply to these research syntheses. Solutions lie in the adoption of alternative designs better suited to make directional and causal inferences in theory effects, particularly longitudinal cross-lagged panel designs and randomized controlled designs, respectively.

Alongside this, concerns have been raised over the use of theory in developing and testing interventions purposed to change behavior. Specifically, researchers have suggested that many interventions do not explicitly match techniques that form intervention content with the targeted theoretical constructs the represent the psychological mechanisms by which the intervention is purported to operate in changing the behavior (e.g., Hagger et al., 2020; Kok et al., 2016; Michie et al., 2018; Sheeran et al., 2017). Further, studies evaluating the efficacy of theory-based interventions do not routinely test these mechanisms, often referred to as intervention mechanisms of action (Hagger et al., 2020a, b). In this section, we outline research that addresses these two key limitations. We initially outline how researchers have employed innovative research synthesis methods to provide robust estimates of theory of planned behavior effects, including syntheses of theory effects in studies adopting longitudinal and randomized controlled designs. We then examine how recent methodological developments could be utilized to test the mechanisms of action of interventions based on the theory. Specifically, we outline how advances in research synthesis methods and the expanding research literature employing appropriate designs enable such syntheses and evaluate their potential to contribute to knowledge on how interventions ‘work’ in changing health behavior.

### Directional and causal inferences

Although most studies testing predictions of the theory of planned behavior in health behavior contexts have employed correlational designs, there is a broadening body of research testing its predictions using designs that enable better directional and causal inferences. For example, researchers have tested theory effects using cross-lagged panel designs that provide better support for directional inferences of theory predictions while controlling for temporal construct change, and in some implementations, intraindividual construct change (e.g., Prati et al., 2014; Reinecke et al., 1996). Such designs also have the added advantage of testing auxiliary hypotheses such as reciprocal effects among constructs through cross-lagged effects. For instance, these designs enable tests of prior behavioral participation effects on social cognition constructs, such as attitudes, in addition to construct effects on subsequent behavior, consistent with received theory (see Liska, 1984). Similarly, researchers have adopted experimental and intervention studies using randomized controlled designs to test the effects of manipulations (e.g., messages, persuasive communication) aimed at activating or changing one or more constructs from the theory on health behavior (e.g., Norman et al., 2018; Sniehotta, 2009). Randomization to conditions provides a better basis for causal inference by minimizing potential influences of extraneous factors that partially or entirely account for the effects^3^. Findings from these studies have provided some convergence in support for theory effects and extend primary research findings adopting correlational designs and prior meta-analyses based on studies adopting correlational designs.

The availability of a critical mass of studies employing longitudinal and randomized designs to test the theory in health behavior since our 2015 meta-analysis (Rich et al., 2015), together with some methodological innovations, has permitted new syntheses of this research. Focusing first on longitudinal designs, we conducted a meta-analysis of 87 longitudinal tests of the theory of planned behavior (Hagger & Hamilton, 2024a). The study also capitalized on innovations in meta-analytic structural equation modelling (e.g., Cheung & Hong, 2017), which allowed us to conduct a cross-lagged panel analysis of theory predictions that included all eligible studies even if they did not report a full panel design or measure all the theory constructs^4^. Findings supported hypothesized theory effects over time while controlling for temporal construct stability and provided little evidence for reciprocal effects among its constructs. Although the analysis provided further robust support for the proposed directional effects of constructs as propose in the theory, the analysis was still based on correlational data and did not employ advanced implementations to analyze panel designs (e.g., random intercept cross-lagged panel analyses) that permit modelling of intraindividual change in addition to temporal change in constructs (e.g., Orth et al., 2021). These are avenues for future research, but analyses adopting random intercept analyses are dependent on gaining access to the raw data sets of included studies, which has, hitherto, presented challenges for research syntheses despite calls for data sharing.

Turning to application of experimental and intervention study designs to test theory predictions, two meta-analyses have been conducted (Sheeran et al., 2016b; Steinmetz et al., 2016). These analyses focused exclusively on studies employing randomized designs including experimental, quasi-experimental, and intervention designs in which participants were randomly assigned to either treatment group exposed to manipulations or techniques aimed at changing one or more theory constructs or a control or comparison group that did not receive the treatment. An important innovation of Sheeran et al. (2016a, b) analysis was the application of a selection method to ensure that included studies sufficiently engaged the targeted theory construct. Specifically, studies had to report statistically significant post-intervention change in a measure of the targeted construct in addition to testing for subsequent change in intentions toward, and actual participation in, the targeted health behavior. This approach has been described as the change meta-analysis method (Sheeran et al., 2021). An important feature of the Steinmetz et al. (2016) analysis was to code studies according to the behavior change methods or techniques used, although they did not segregate their analysis into studies targeting change in each individual theory construct. Sheeran et al. (2016a, b) analysis indicated unique change in health behavior intentions and behavior in studies that targeted change in attitudes, intention, and behavior, with effect sizes on intentions larger than effect sizes on behavior, consistent with previous analyses (e.g., Webb & Sheeran, 2006). Similarly, Steinmetz et al. (2016) identified changes in theory constructs (attitude, subjective norm, and perceived behavioral control) and intentions and behavior in interventions based on the theory. They also identified persuasion, increasing skill use, and motivational strategies were most effective in changing selected theory constructs and intentions, but with few differences on behavior. Taken together, findings from these analyses provide the most robust evidence to date for causal effects of theory constructs on intention and behavior change in the theory of planned behavior, and point to candidate methods that may be effectively employed in behavior change interventions based on the theory. The syntheses also provide evidence that interventions with a basis in theories like the theory of planned behavior can lead to effective behavior change.

### Mechanisms of action of theory-based interventions

Although researchers have used theories such as the theory of planned behavior as a basis for interventions to change health behavior, studies evaluating these interventions have not routinely tested whether the methods or techniques that form the content of interventions operate to change behavior as specified in theory (Hagger et al., 2020a, b; Sheeran et al., 2017). Specifically, researchers have not been sufficiently explicit or consistent in describing how the techniques used match the targeted theory construct (Kok et al., 2016; Michie et al., 2018). Furthermore, researchers adopting randomized controlled studies to test intervention effects have not tended to include measures or analyses to evaluate whether technique exposure leads to change both the theoretical constructs and behavioral outcome (Hagger, 2025). Advances in research in the science of behavior change has led to the development of systematic definitions and descriptions of the techniques used in behavior change interventions organized into structured classifications – referred to as taxonomies and ontologies (e.g.,Marques et al., 2023). Alongside this, there have been evidence-based efforts to match these techniques with theory-based constructs that represent the mental processes proposed to be changed or activated by the technique (Carey et al., 2019; Connell et al., 2019; Kok et al., 2016), with accompanying protocols for researchers to do so (Birk et al., 2023; Johnston et al., 2021; Michie et al., 2018). Finally, researchers have also specified the kinds of designs and analyses necessary to provide fit-for-purpose evidence to support the proposed process by which such techniques operate to change behavior by engaging the targeted theory-based construct, known as the intervention mechanism of action (Birk et al., 2023; Hagger, 2025; Sheeran et al., 2017). Taken together, this research has provided the clearest guidance yet on how researchers can test how their interventions work in changing behavior.

However, meta-reviews of behavior change interventions have indicated that evidence testing theory-based mechanisms of action is relatively sparse (Hagger et al., 2020a, b; Hennessy et al., 2020). As a consequence researchers have been comparatively limited in their capacity to conduct syntheses of intervention mechanisms of action such that cumulative evidence on such mechanisms is a recognized need (Hennessy et al., 2022). Such an evidence base will provide interventionists with knowledge of the techniques likely to have efficacy in changing behavior, how they operate, and the conditions that may determine their efficacy (e.g., behavior, context, population characteristics). The meta-analyses of experimental and intervention research based on the theory of planned behavior outlined in the previous section provide important advances that contribute to this evidence based insofar as their efficacy, their engagement of the theoretical constructs, and the techniques used (Sheeran et al., 2016b; Steinmetz et al., 2016). However, they do not provide formal tests of mechanisms of action, that necessitate studies reporting sufficient data to conduct mediation analyses to confirm indirect effects of strategies or manipulations on change in behavioral outcomes through change in the theory construct proposed to be implicated in the change. Recent syntheses have advanced this research by providing such analyses. For example, Rhodes et al. (2021) demonstrated indirect effects of multiple techniques on physical activity behavior change mediated by constructs from multiple theories and Sheeran et al. (2020) demonstrated indirect effects of strategies from self-determination theory, a humanistic, needs-based theory of motivation, on health behavior change mediated by the autonomy and competence constructs from the theory. Both analyses revealed indirect effects that yielded cumulative evidence in support of their respective theory-based mechanism of action.

However, both analyses were limited insofar as the data used did not account for change in the behavioral outcome resulting from change in the theory-based construct itself, that is, the mediator (for a discussion see Bullock & Green, 2021). Such effects were, instead, based on correlational data (Sheeran et al., 2021). In future, meta-analyses need to conduct syntheses of intervention studies that report effect size data in which the intervention techniques used lead to demonstrable change in both the behavior and the targeted theoretical construct post-intervention, and, importantly, that change in the construct leads to change in the behavior post-intervention. This essentially extends Sheeran et al.’s (2021) change meta-analysis method to a full mediation analysis. However, few studies provide sufficient data to compute such analyses. There have been calls for researchers to report such data and methods to identify and archive such data that may feed into future evidence syntheses (Hagger, 2025; Hennessy et al., 2022). Such endeavors will serve to iteratively develop a cumulative evidence base of intervention mechanisms of action in health behavior research.

## Summary and conclusion

Our 2015 meta-analysis of the theory of planned behavior provided cumulative evidence for associations between theory constructs and health-related intentions and behavior in populations with chronic illness (Rich et al., 2015). Our analysis contributed further to the evidence of psychological determinants of health behavior together with other meta-analyses of the theory (e.g., Albarracín et al., 2001; Hagger et al., 2002; Hamilton et al., 2020; McEachan et al., 2011). Since that time, new meta-analyses of research applying the theory in health behavior contexts have capitalized on theoretical, methodological, and analytic innovations to address limitations and fill evidence gaps of the prior syntheses. These analyses have made important contributions to knowledge on health behavior determinants research and behavior change interventions based on the theory. In this review, we summarized the findings of these meta-analyses, evaluated their contribution, highlighted arising limitations and evidence gaps, and suggested avenues for research.

We began by highlighting limitations and gaps in knowledge on the theory and how prior research syntheses have fallen short of providing sufficient evidence to address them. Specifically, we outlined theory predictions that our study, and other contemporary meta-analyses, had not sufficiently addressed or tested, including (a) moderating effects of perceived behavioral control and construct correspondence and the mediated effects of indirect and direct measures of theory constructs on direct theory constructs, intention and behavior; (b) effects of variables and constructs representing sociostructural and intrapersonal health behavior determinants; and (c) effects of constructs representing non-conscious processes. We highlighted the contributions of new meta-analyses that capitalized on methodological innovations and evidence proliferation that lend support for perceived behavioral control moderating effects (Hagger et al., 2022), and effects of sociostructural variables (Hagger & Hamilton, 2021; McAnally & Hagger, 2023), and constructs representing non-conscious processes (e.g., habit), on behavior (Hagger et al., 2023). We highlighted how these syntheses have also shed light on proposed theory-based assumptions and mechanisms (e.g., mediation of sociostructural variable effects on behavior by theory constructs; conditions determining relative contributions habit and intention on behavior). We also highlighted the need for additional syntheses addressing further theory-relevant limitations, and offered our currently in-progress meta-analyses of the theory incorporating personality traits (Hagger, 2018) and implicit attitudes (Phipps et al., 2024) as examples and discussed their implications.

Next, we outlined key methodological issues that placed limits on the inferences that could be drawn from prior meta-analyses of the theory in health behavior contexts: (a) including inferences of direction and cause in theory effects; and (b) tests of intervention mechanisms of action in the theory. We highlighted recent syntheses that capitalized on recent advances in methods and advances in data availability that partially addressed these limitations. Our recent meta-analysis of longitudinal research that tested directional and reciprocal effects in the theory (Hagger & Hamilton, 2024a), and two recent meta-analyses of experimental and intervention research (Sheeran et al., 2016b; Steinmetz et al., 2016), were presented as examples of these innovations. We also highlighted the limitations of these analyses, and proposed recommendations for future meta-analyses testing the mechanisms of action of interventions based on the theory. Specifically, we suggested modifications to the mediation analyses used to test mechanisms of action of behavior change techniques in other theories as starting points for these proposed syntheses and how they might be modified to do so (Rhodes et al., 2021; Sheeran et al., 2020).

Overall, the current review of new meta-analyses conducted since our 2015 analysis are indicative of the considerable progress that has been made in cumulative evidence on applications of theory in health behavior contexts. However, we have also highlighted ongoing deficiencies and gaps in available syntheses and signaled need for new syntheses to address them. We have offered our ongoing work and suggestions of new syntheses, particularly meta-analyses of experimental and intervention research and tests of mechanisms of action of behavior change, as possible avenues for future research and expect them to inform an agenda for new syntheses that advance knowledge on the theory in health behavior contexts.

## Data Availability

Not applicable.

## Footnotes

1 It should be noted that recent commentaries have identified limitations in the use of some implementations of these analytic approaches in meta-analyses of the theory of planned behavior and offered potential solutions (e.g., Cheung & Hong, 2017; Hagger & Hamilton, 2024b; Jak & W.-L. Cheung, 2024).

2 Ajzen (1991) referred to the broad, summary measures of the attitude, subjective norm, and perceived behavioral control constructs as *direct* construct measures while measures of the specific behavioral, normative, and control beliefs were referred to as *indirect* measures. Here were refer to the direct construct measures as *global* measures and the indirect measures as *belief-based* measures in order to avoid confusion when discussing direct and indirect (mediated) effects of these constructs in tests of the theory.

3 It should be noted that such designs do no entirely obviate issues relating to causal inference and other considerations need to be taken into account, for a review see Rohrer (2024).

4 For a discussion of analytic issues including meta-analyzing data from studies where the numbers of effect sizes in the analyzed matrix of correlations among theory constructs vary and its use to estimate novel models, see Hagger and Hamilton (2024b).
